# APN-mediated phosphorylation of BCKDK promotes hepatocellular carcinoma metastasis and proliferation via the ERK signaling pathway

**DOI:** 10.1038/s41419-020-2610-1

**Published:** 2020-05-26

**Authors:** Mengying Zhai, Zixia Yang, Chenrui Zhang, Jinping Li, Jing Jia, Lingyi Zhou, Rong Lu, Zhi Yao, Zheng Fu

**Affiliations:** 1 Department of Immunology, Key Laboratory of Immune Microenvironment and Disease of the Educational Ministry of China, Tianjin Key Laboratory of Cellular and Molecular Immunology, School of Basic Medical Sciences, Tianjin Medical University, 300070 Tianjin, China; 2 Tianjin Kangzhe Pharmaceutical Technology Development Company, Ltd., 300042 Tianjin, China; 3 2011 Collaborative Innovation Center of Tianjin for Medical Epigenetics, Tianjin Medical University, 300070 Tianjin, China

**Keywords:** Phosphoproteins, Metastasis, Tumour biomarkers

## Abstract

Hepatocellular carcinoma (HCC) is one of the most prevalent human malignancies worldwide and has high morbidity and mortality. Elucidating the molecular mechanisms underlying HCC recurrence and metastasis is critical to identify new therapeutic targets. This study aimed to determine the roles of aminopeptidase N (APN, also known as CD13) in HCC proliferation and metastasis and its underlying mechanisms. We detected APN expression in clinical samples and HCC cell lines using immunohistochemistry, flow cytometry, real-time PCR, and enzyme activity assays. The effects of APN on HCC metastasis and proliferation were verified in both in vitro and in vivo models. RNA-seq, phosphoproteomic, western blot, point mutation, co-immunoprecipitation, and proximity ligation assays were performed to reveal the potential mechanisms. We found that APN was frequently upregulated in HCC tumor tissues and high-metastatic cell lines. Knockout of APN inhibited HCC cell metastasis and proliferation in vitro and in vivo. Functional studies suggested that a loss of APN impedes the ERK signaling pathway in HCC cells. Mechanistically, we found that APN might mediate the phosphorylation at serine 31 of BCKDK (BCKDK^S31^), promote BCKDK interacting with ERK1/2 and phosphorylating it, thereby activating the ERK signaling pathway in HCC cells. Collectively, our findings indicate that APN mediates the phosphorylation of BCKDK^S31^ and activates its downstream pathway to promote HCC proliferation and metastasis. Therefore, the APN/BCKDK/ERK axis may serve as a new therapeutic target for HCC therapy, and these findings may be helpful to identify new biomarkers in HCC progression.

## Introduction

Hepatocellular carcinoma (HCC) represents the most common type of primary liver cancer with high morbidity and high mortality. As one of the most prevalent global human malignancies, the number of new HCC cases and related deaths worldwide in 2018 were ~841,000 and 781,000, respectively^1^. More than 50% of liver cancer cases occur in China. Despite various advances in diagnosis and treatment, the high probability of metastasis makes its prognosis far from satisfactory^2–5^. Thus, understanding the molecular mechanisms underlying HCC development and metastasis is an urgent need for identifying new therapeutic targets and developing new approaches to reduce HCC mortality.

Aminopeptidase N (APN/CD13, EC3.4.11.2) is a Zn^2+^-dependent membrane-bound peptidase that is widely distributed in many mammalian tissues, such as the intestine, kidney, liver, and central nervous system^6^. APN can cleave peptides to release N-terminal neutral amino acids such as Ala, Phe, and Leu^7^. Originally discovered during the search for specific markers for human leukemia classification, APN is now considered a multifunctional (“moonlighting”) protein with a hydrolytic ability. APN is involved in the activation or degradation of bioactive peptides, degradation of extracellular matrix, signal transduction, and antigen presentation and serves as a receptor for some human viruses (e.g., coronaviruses)^8^. Although APN has been identified as a candidate HCC stem cell marker^9,10^, the exact mechanism of APN in the proliferation and metastasis of HCC is still unclear.

The reversible phosphorylation of various proteins regulates function, subcellular localization, complex formation, and degradation of these signaling molecules. As a result of all of these modifications, the signal transduction network is mediated in cells. It is estimated that between 30% and 65% of all proteins may be phosphorylated, some multiple times^11,12^. Phosphorylated proteins and mediators of these modifications may be useful molecular cancer markers that are invaluable for the diagnosis, prognosis prediction and discovery of therapeutic targets. Branched-chain α-ketoacid dehydrogenase kinase (BCKDK) is a member of a distinctive family of mitochondrial protein kinases that is similar to prokaryotic histidine kinases, whose function is to inactivate BCKD complexes by phosphorylation, thereby preventing the catabolism of these essential regulatory metabolites^13,14^. BCKDK plays an important role in many serious human diseases, such as Kaufman oculocerebrofacial syndrome (KOS)^15^, obesity-associated insulin resistance (IR)^16^, dilated cardiomyopathy (DCM)^17^, and epilepsy in autism^18^. However, there is little research linking BCKDK and cancer, and the relationship between BCKDK and HCC is unclear.

In this study, we demonstrated that knockout of APN inhibits the migration, invasion and proliferation of HCC cells and suppresses HCC metastasis and growth in vivo. Functional studies suggested that loss of APN impedes the ERK signaling pathway in HCC cells. Mechanistically, we found that APN might mediate the phosphorylation of serine at position 31 of BCKDK, then promote BCKDK binding and phosphorylating ERK1/2, thereby activating the ERK signaling pathway in HCC cells. Taken together, these data support a role for APN as a promoting factor in HCC growth and metastasis, implying its potential as a therapeutic target.

## Materials and methods

### Cell lines and culture

HCC cell lines (SK-HEP-1, RRID: CVCL_0525; Huh7, RRID: CVCL_0336; HepG2, RRID: CVCL_0027; and Hep3B, RRID: CVCL_0326) and human embryonic kidney cell line 293T (RRID: CVCL_0063) were purchased from the American Type Culture Collection (ATCC, Manassas, VA, USA). Several other HCC cell lines (Huh6, RRID: CVCL_4381; MHCC97H, RRID: CVCL_4972; and BEL-7402, RRID: CVCL_5492) were obtained from the Cell Bank of the Chinese Academy of Sciences (Shanghai, China). The normal liver cell THLE-3 (RRID: CVCL_3804) was purchased from the Beina Chuanglian Biotechnology Institute (Beijing, China).

All HCC cell lines and 293T cell were maintained in Dulbecco’s Modified Eagle’s Medium (DMEM, Gibco, Gaithersburg, MD, USA) supplemented with 10% fetal bovine serum (FBS, Gibco). THLE-3 cell line were cultured in Roswell Park Memorial Institute-1640 (RPMI-1640, Gibco) supplemented with 10% FBS. All cells were grown in a humidified incubator with 5% CO_2_ at 37 °C, routinely were authenticated by examination of morphology and growth characteristics, and were tested using MycoAlert detection Kit (Lonza, Cologne,Germany) for mycoplasma contamination. Only mycoplasma negative cells were used for experiments.

### APN expression and activity assay

To evaluate the expression of APN on the cell surface, HCC cells and normal hunman liver cell line THLE-3 were incubated with APC-conjugated anti-human APN/CD13 (Biolegend, San Diego, CA, USA) or APC Mouse IgG1, κ Isotype Ctrl (Biolegend) antibodies for 30 min at 4 °C. Then, the cells were analyzed by using a FACS Canto^TM^ II flow cytometer (BD Biosciences, San Jose, CA, USA).

APN activities in different cell lines were estimated by measuring the hydrolysis of a fluorescent substrate derived from alanine conjugated to 7-amido-4-methylcoumarin (Ala-AMC, Bachem, Budendorf, Switzerland), as described previously^19^. The THLE-3 cell line was used as control.

### RNA extraction and reverse transcription-quantitative PCR (RT-qPCR)

Total RNA was extracted using the TRIzol Reagent (Ambion, Austin, TX, USA) and then reverse transcribed to cDNA using the First Strand cDNA Synthesis Kit (Thermo Fisher Scientific) following the protocol of the suppliers. The expression levels of target genes were analyzed by quantitative real-time PCR using SYBR green mix (Applied Biosystems, USA). The data were calculated by the 2^–ΔΔCt^ method and are representative of at least three independent experiments. The THLE-3 cell line was used as control. β-actin was used as a housekeeping gene. The primers used in this study are listed in Supplementary Table S1.

### Migration and invasion assays

HCC cells were starved overnight, detached with trypsin, and resuspended in serum-free medium. For the transwell migration assay, 5 × 10^4^ HCC cells were placed in the upper chamber of each insert (pore size 8 micros (μm), Corning, NY, USA) containing the noncoated membrane. For the invasion assay, cells were placed in the upper chamber of each insert that was coated with 100 microliters (μl) of Matrigel (BD biosciences), which was diluted 1:50 with serum-free medium. Then, medium supplemented with 10% fetal bovine serum was added to the lower chambers. After 24–48 h (h) of incubation, cells in the upper chamber were removed by wiping with a cotton swab. Cells on the lower surface in chamber were fixed with 4% formaldehyde (Sigma-Aldrich, St. Louis, MO, USA) for 1 h and stained using 0.1% crystal violet for 15 minutes (min). Finally, the filters were washed three times in phosphate-buffered saline (PBS), and images were taken using a Nikon ECLIPSE 90i microscope (Tokyo, Japan). Cell numbers were counted in five randomly selected fields (200×) from each well. The ERK inhibitor (HY-50846) used in transwell assays was obtained from MedChem Express (Monmouth Junction, NJ, USA), and epidermal growth factor (EGF) was purchased from Gibco (Grand Island, NY, USA).

### Tissue microarray and immunostaining

Tissue microarray slides containing 180 specimens from 90 HCC patients were obtained from Outdo Biotech (Shanghai, China). The information of tumor specimens can be found in Supplementary Table S2. Immunostaining was performed with a rabbit anti-CD13/APN antibody from Cell Signaling Technology (CST, Danvers, MA, USA) as described previously^20^. Images of stained sections were taken with an Olympus microscope (Tokyo, Japan). To evaluate the immunostaining quantification, we analyzed the integral optical density (IOD) and positively stained area of micrographs via an image analysis workstation (Image Pro Plus 6.0, Media Cybernetics). The expression levels of APN were recorded by calculating the ratio of IOD to area as previously described^21^. The study was approved by the Ethics Committee of the Tianjin Medical University, and informed consent was obtained from all patients.

### Plasmids, transfection, and generation of stable cell lines

The plasmids for ectopic overexpression of the target human proteins Flag-tagged ERK1, Flag-tagged ERK2, Flag-tagged BCKDK, Myc-tagged ERK1, Myc-tagged ERK2 and 6xMyc-tagged BCKDK were purchased from Genechem Corporation (Shanghai, China). Nonphosphorylatable S31A and phosphomimetic S31D mutants of the BCKDK protein were generated with the Fast Mutagenesis System Kit (TransGen Biotech, Beijing, China) by using primer pairs BCKDK(S31A)-F/BCKDK(S31A)-R and BCKDK(S31D)-F/BCKDK(S31D)-R, respectively. Primer sequences are listed in Supplementary Table S3. Transfection was performed using the Lipofectamine 3000 reagent (Invitrogen, Carlsbad, CA, USA) following the manufacturer’s instructions.

Clustered regularly interspaced short palindromic repeat (CRISPR) genome editing was used to generate APN knockout (KO) HCC cell lines, as described previously^22^. APN sgRNAs were cloned into lenti-CRISPR V2 vectors (Addgene, Watertown, MA, USA). The sgRNAs were designed using the CRISPR Design Tool (http://crispr.mit.edu; Zhang Laboratory, Massachusetts Institute of Technology, Cambridge, MA, USA) and purchased from Sangon Biotech (Shanghai, China). The sgRNA sequences are listed in Supplementary Table S4.

Lentiviruses for generating stable cell lines that express ectopic full-length human APN, human BCKDK^S31D^ or firefly luciferase were constructed by Genechem Corporation (Shanghai, China). HCC cells were infected with lentivirus in the presence of 10 micrograms per milliliter (μg/ml) polybrene (Sigma-Aldrich). At 48 h after the infection, the medium was changed, and cells were selected with puromycin (2 milligram per milliliter (mg/ml), Sigma-Aldrich).

### Antibodies and western blotting

Whole-cell protein extraction and western blotting were performed as described previously^23^. Information on all primary antibodies used in this study is provided in Table S5. Antibody binding was revealed using an HRP-conjugated anti-rabbit IgG or anti-mouse IgG (Sigma-Aldrich). Antibody complexes were detected using Immobilon Western Chemiluminescent HRP Substrate (Merck Millipore, Billerica, MA, USA).

### Animal studies/in vivo experiments

Six-week-old female NOD/SCID (RRID: IMSR_ARC:NODSCID) mice were purchased from the Academy of Military Medical Science (Beijing, China). All experimental procedures involving animals in this study were reviewed and approved by the Animal Ethics Committee of Tianjin Medical University. Six or five animals randomly assigned per group were used in each experiment. Sample size estimate was based on xenograft assays from literatures. The measurement and data processing were done with blinding. Except the survival assay, all animals were sacrificed at the end of the experiment and included into the analysis.

For subcutaneous xenograft experiments, SK-HEP-1 APN knockout cells (SK-KO) and SK-HEP-1 control cells (SK-Con) were transfected with lentiviruses to express firefly luciferase and injected subcutaneously into the right flanks of NOD/SCID mice (5 × 10^6^ cells per mouse). The mice were euthanized six weeks after injection, and tumors were removed and the volume was calculated using the following formula: tumor volume (cubic millimeters (mm^3^)) = (length × width^2 ^× *π* × 1/6).

For the in vivo liver metastasis model, HCC cells were implanted into the livers of NOD/SCID mice via injection into the spleen^24^. A small abdominal incision was made in the left flank to isolate the spleen and extract it. Luciferase-expressing cells (SK-Con and SK-KO) were injected into the spleen (1 × 10^6^ cells per mouse). Ten min later, the blood vessels of the spleen were ligated, the spleen was removed, and the abdominal wall was sutured. Seven weeks after the injection, the mice were sacrificed, and the livers, lungs, and kidneys were isolated and examined for metastasis with an IVIS Spectrum scanner.

For the liver orthotopic xenograft model, NOD/SCID mice were anesthetized using 1.5% inhaled isoflurane, and the left lobe of the liver was exposed through an upper midline laparotomy. Luciferase-expressing cells (SK-Con, SK-KO and SK-KO overexpressing BCKDK^S31D^) in 30 μl of culture medium containing 50% Matrigel (BD Biosciences) were injected into the left lobe (2 × 10^6^ cells per mouse). Eight weeks after orthotopic HCC cell injection, all mice were euthanized. The livers, lungs, spleens, and kidneys of mice were isolated, and the bioluminescent signals of these organs were observed using an IVIS Spectrum scanner to verify tumor establishment and metastasis.

For the survival assay, SK-KO cells and SK-Con cells were injected into the livers of NOD/SCID mice (2 × 10^6^ cells per mouse). Mice injected with cells were fed normally until their death, and the date of death was recorded.

### Apoptosis assays

For analysis of apoptosis, 5 × 10^5^ cells/dish were seeded into 60-mm dishes in complete media overnight and then treated with cisplatin (0.02 mg/ml, Sigma-Aldrich) for 24 h. Apoptotic rates were assessed with the Annexin V Apoptosis Detection Kit (BD Pharmingen, San Diego, CA, USA) as instructed by the manufacturer. Apoptotic cells were identified by a FACS Canto^TM^ II flow cytometer (BD Biosciences). Data were analyzed using FlowJo software (Version X; TreeStar, Ashland, OR, USA).

### Colony formation assay

For the cell colony formation assay, treatment group cells and control cells were seeded at a density of 400 cells/well in a 2 ml volume of 6-well plates. The media was changed every 3 days. After 9–12 days of culture, the cells were washed with PBS and fixed with 4% paraformaldehyde. The fixed cells were stained with freshly prepared crystal violet for 15 min. The number of colonies formed by cells was observed by microscopy (IX73, Olympus).

### RNA sequencing (RNA-seq)

Total RNA was extracted with TRIzol according to the manufacturer’s instructions and delivered to Beijing Genomics Institute (Beijing, China) for RNA-seq and analyses. The details can be found in the supplementary materials and methods.

### Phosphoproteomic analysis

To perform APN phosphoproteomic screens, SK-HEP-1 cells with or without APN were collected and delivered to Genechem Corporation (Shanghai, China) for phosphoproteomic analysis. The details can be found in the supplementary materials and methods.

### Phospho-antibody production

The anti-BCKDK (S31-P) antibody was produced according to the method described previously^25^. Briefly, a phosphorylated BCKDK peptide (RARS(p)TSATDTgg-c) was synthesized and conjugated to KLH. Rabbits were immunized by this peptide. A phosphor-peptide column was used for affinity-purification of antiserum, and further purification was performed by a nonphosphorylated peptide. The specificity and titer of the purified anti-BCKDK (S31-P) antibody was tested by western blot and ELISA analysis.

### Proximity ligation assay (PLA)

Interactions between BCKDK and ERK1 or BCKDK and ERK2 in SK-HEP-1 cells were detected by Duolink® In Situ Red Starter Kit Mouse/Rabbit (Sigma-Aldrich). Cells were first cultivated on round cover glasses. Slides were blocked and incubated with primary antibodies targeting the interacting proteins after fixation and permeabilization. The antibodies are provided in Table S5. Then, the cultures were washed and incubated with PLA probes (anti-goat MINUS and anti-rabbit PLUS) for 1 h at 37 °C. Ligation and amplification were processed according to the manufacturers’ instructions in the presence of polymerase. Cell nuclei were stained using Duolink® In Situ Mounting Medium with DAPI. Images were taken 15 min after staining with a fluorescence microscope (IX73, Olympus).

### Co-Immunoprecipitation

The HCC cells transfected with the abovementioned plasmids were harvested and lysed in cell lysate buffer (50 millimoles per liter (mM) Tris-HCl, pH 7.4; 150 mM NaCl; 1 mM EDTA; and 1% NP40) with complete protease inhibitors. Two milligrams of protein was incubated with anti-Flag agarose beads (M2, Sigma-Aldrich) at 4 °C overnight to pull down the corresponding exogenous proteins. For co-immunoprecipitation of the endogenous proteins, anti-ERK1, anti-ERK2, and anti-BCKDK antibodies were preincubated with protein A/G agarose (Pierce, Rockford, IL, USA) for 8 h. Normal rabbit IgG (CST) and Normal mouse IgG (Santa Cruz) were used as controls. Proteins were immunoprecipitated with the sepharose-antibody complexes on a rotator at 4 °C overnight. The beads were washed five times with cell lysate buffer and collected by centrifugation at 1000 grams (g), and the immunoprecipitates were subjected to Western blot with the corresponding antibodies. The primary antibodies used for immunoprecipitation are provided in Table S5. Light chain-specific mouse anti-rabbit (#93702, CST) and rabbit anti-mouse (#58802, CST) IgG were used as secondary antibodies for immunoblotting.

### Statistical analysis

Data from biological triplicate experiments were expressed as mean ± standard deviation (SD) or median with interquartile range (IQR) for continuous variables. All tests of significance were two-sided. The Kolmogorov–Smirnov test was used to verify a normal distribution. The *F*-test was used to verify similar variances. Student’s *t*-test was used to analyze statistical significance between the two groups with normal distribution data. All quantitative data in the present experiments were performed three times. For nonnormal distribution data, significant differences between the two groups were analyzed by Wilcoxon signed-rank test. The Mann–Whitney *U*-test and Kruskal–Wallis test were used to analyze data from two or more independent samples. The survival curve was assessed using a log-rank test. Statistical analyses were performed using GraphPad Prism 7.0 software (La Jolla, CA, USA) and IBM SPSS Statistics 19.0 software (IBM, Armonk, NY, USA). Statistical significance was specified as *p < 0.05, **p < 0.01 or ***p < 0.001, ****p < 0.0001.

### Data availability

The mass spectrometry phosphoproteomics data have been deposited to the ProteomeXchange Consortium via the PRIDE^26^ partner repository with the dataset identifier PXD016468.

Raw data of the RNA-seq results are available at the Sequence Read Archive (SRA) data repository (https://www.ncbi.nlm.nih.gov/sra/) with the accession number of PRJNA591769.

## Results

### APN expression is upregulated in HCC tissues and high-metastatic cells, and correlated with HCC progression

**Fig. 1:**
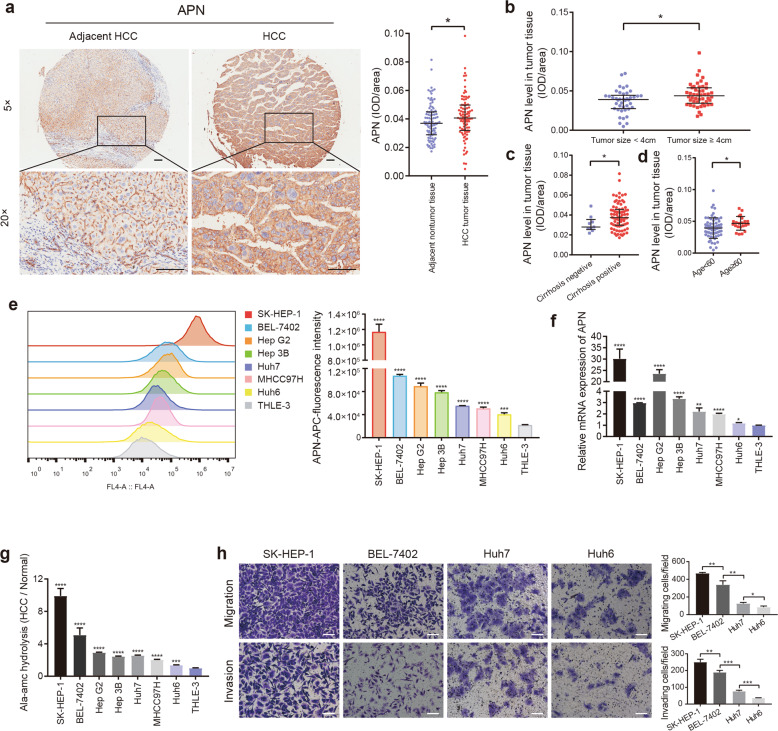
APN expression is upregulated in HCC tissues and high-metastatic HCC cells. **a** APN levels in HCC tumor tissues and adjacent nontumor tissues were analyzed by immunohistochemistry (*n* = 90 in each group). Scale bar: 100 μm. Median with IQR, *p < 0.05, Wilcoxon signed-rank test. **b–d** APN expression in HCC tissues was correlated with the tumor size, cirrhosis and age of patient. Median with IQR, *p < 0.05, Mann–Whitney *U-*test. **e** Flow cytometry revealed APN content in different HCC cell lines and normal human liver cell line THLE-3. **f** Expression of APN in different cell lines were measured by RT-PCR assay. The human normal liver cell line THLE-3 was used as control. **g** Enzyme activity of APN was measured based on the levels of Ala-AMC hydrolysis versus control. The THLE-3 cell line was used as control. **h** Transwell assays showed migration and invasion of different HCC cells. Scale bar: 100 μm. All quantitative data in the present experiments were performed three times. Mean ± SD, *p < 0.05, **p < 0.01, ***p < 0.001, ****p < 0.0001, Student’s *t*-test.

To investigate the role of APN in HCC progression, we analyzed its expression levels in a tissue microarray containing 90 samples of HCC patients by immunostaining. The expression levels of APN were significantly higher in HCC tumor tissues than in adjacent nontumor tissues (Fig. 1a). Moreover, a clinical pathological analysis of the tumor tissues indicated that higher APN expression was significantly positively associated with tumor size (Fig. 1b), cirrhosis occurrence (Fig. 1c) and patient age (Fig. 1d). To further demonstrate that APN is associated with HCC progression, we measured the expression levels of APN in seven HCC cell lines and a normal human liver cell line using flow cytometry and RT-qPCR. APN expression showed significant differences in different HCC cell lines, and APN levels in all HCC cell lines are significantly higher than that of normal liver cell (Fig. 1e, f). The trend of APN enzyme activity detection is consistent with its expression level in different cell lines (Fig. 1g). Then, we selected four HCC cell lines with different APN levels and evaluated their metastatic ability by a transwell assay (Fig. 1h). We found that high-APN HCC cells have a greater metastatic potential than low-APN HCC cells. These results supported the notion that increased APN expression is associated with human liver cancer metastasis and proliferation.

### APN promotes HCC cell metastasis in vitro and in vivo

**Fig. 2:**
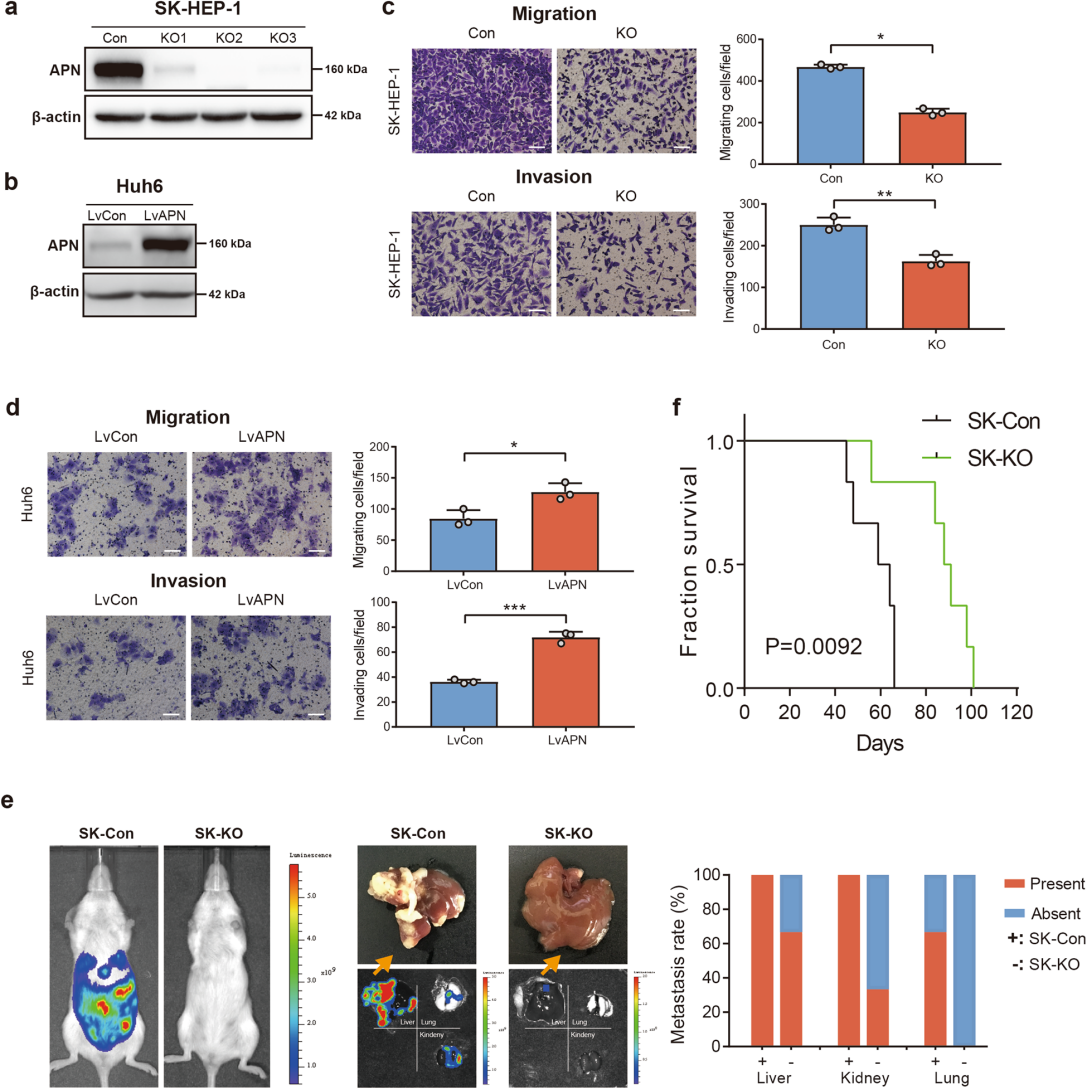
Alteration of APN can regulate liver cancer cell metastasis in vitro and in vivo. **a** After CRISPR genome editing, APN was knockout in SK-HEP-1 cells. **b** After lentivirus transfection, APN was overexpressed in Huh6 cells. **c** Transwell assays were used to evaluate the migration and invasion of SK-HEP-1 cell after knockout of APN. **d** Transwell assays were used to evaluate the migration and invasion of Huh6 cell after APN overexpression. Scale bar: 100 μm. The experiments were performed three times. Mean ± SD, *p < 0.05, **p < 0.01, ***p < 0.001, Student’s *t*-test. **e** Representative bioluminescence imaging of intrahepatic metastatic model derived from splenic implantation with luciferase-labeled SK-Con and SK-KO cells (*n* = 6 in each group). Representative gross specimens of metastatic lesions in liver, lung, and kidney. Occurance rates of metastasis in different organ metastases are represented by histogram. **f** Kaplan–Meier plot showing overall survival of mice injected with SK-Con and SK-KO cells via liver (*n* = 6 in each group). *P* = 0.0092, log-rank test.

To ascertain the function of APN in HCC progression, we selected SK-HEP-1 cells with high-APN expression and Huh6 cells with low-APN expression for loss- and gain-of-function studies. APN was knocked out in SK-HEP-1 cells by CRISPR genome editing, and APN sgRNA-KO2 was verified and chosen as the best candidate for knocking out APN expression in SK-HEP-1 cells (Fig. 2a). Overexpression of APN in Huh6 cells was accomplished by lentivirus (LvAPN) transfection (Fig. 2b). Compared with control cells (SK-Con), the migration and invasion ability of APN knockout cells (SK-KO) was greatly reduced (Fig. 2c). Corresponding to this loss-of-gain phenotype, overexpression of APN significantly promoted the migration and invasion of Huh6 cells (Fig. 2d).

To investigate the effect of APN on the metastasis ability of HCC cells in vivo, we performed a liver metastasis experiment with splenectomy. The spleen is a highly vascularized structure from which tumor cells appear to readily travel into the liver via the splenic and portal veins. The mice injected with APN knockout SK-HEP-1 cells (SK-KO) exhibited fewer and smaller liver metastatic nodules and a lower rate of tumor metastasizing to other organs than did the SK-Con group (Fig. 2e). Moreover, we applied a liver orthotopic xenograft model to simulate the progression of clinical liver cancer. SK-Con and SK-KO cells were implanted into the livers of NOD/SCID mice, respectively. The SK-KO group showed longer survival times (Fig. 2f). These results provide further evidence that APN is involved in promoting HCC invasion and metastasis.

### APN knockout inhibited proliferation of HCC cells in vitro and in vivo

**Fig. 3:**
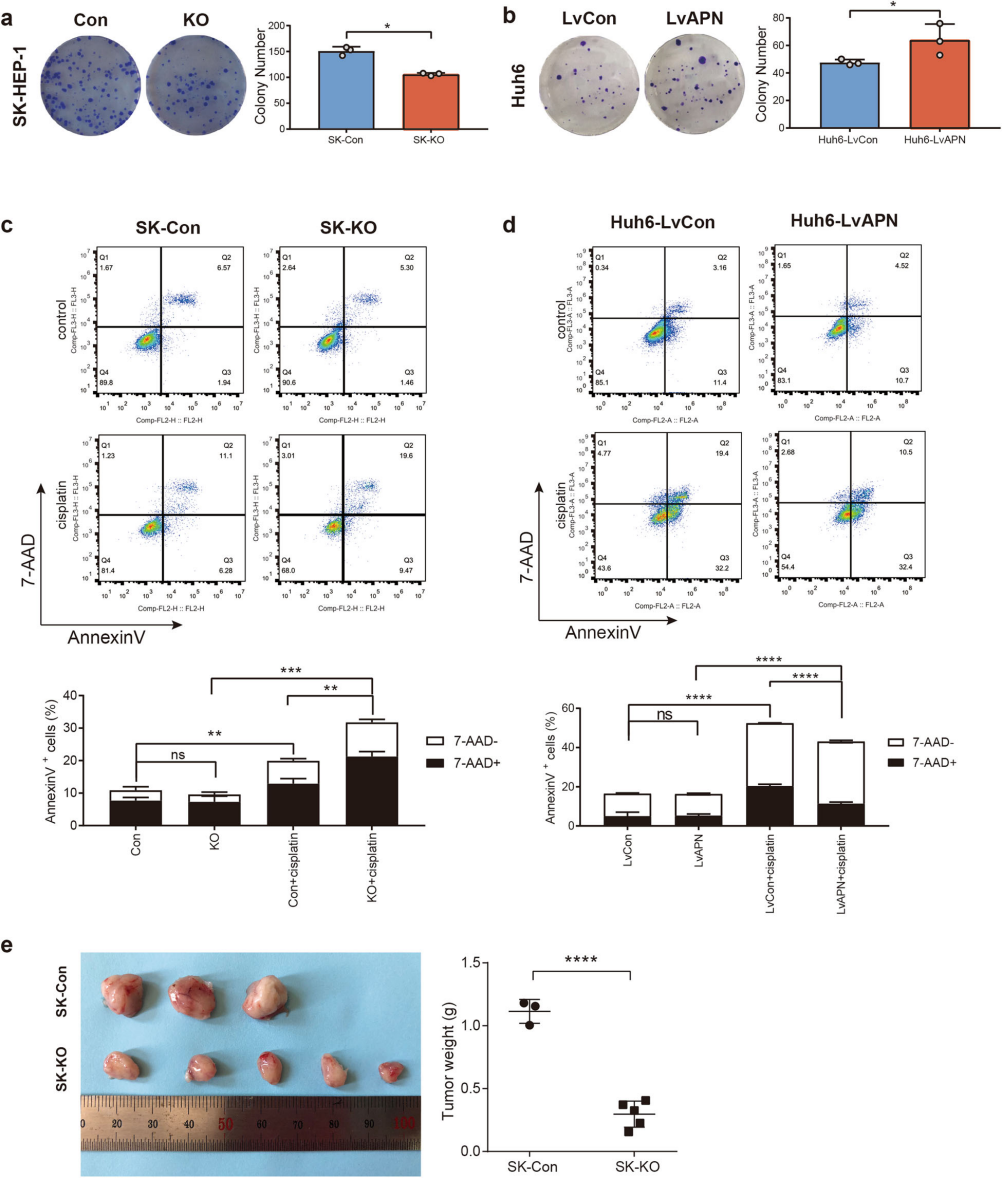
Alteration of APN can regulate HCC cells proliferation in vitro and in vivo. **a** Colony formation assay showed effect of APN knockout on cell proliferation in SK-HEP-1 cells. **b** Colony formation assay showed effect of APN overexpression on cell proliferation in Huh6 cells. Mean ± SD, *p < 0.05, Student’s *t*-test. **c** SK-Con and SK-KO cells were treated with or without Cisplatin, and apoptosis of indicated cells were detected using flow cytometry. **d** Huh6-LvCon and Huh6-LvAPN cells were treated with or without Cisplatin, and apoptosis of indicated cells were detected using flow cytometry. All quantitative data in the present experiments were performed three times. Mean ± SD; ns non-significant; **p < 0.01, ***p < 0.001, ****p < 0.0001; Student’s *t*-test. **e** Luciferase-labeled SK-Con and SK-KO cells were subcutaneously injected into NOD/SCID mice (*n* = 5 in each group, two mice in the control group died during the experimental observation). Tumors were harvested and tumor weight was measured at the end of the experiment. Mean ± SD, ****p < 0.0001, Student’s *t*-test.

Colony formation assays were used to show that depleting the APN level significantly attenuated the growth of HCC cells in vitro. The SK-KO cells formed fewer and smaller colonies than the SK-Con cells (Fig. 3a), while in the cells overexpressing APN (Huh6-LvAPN), the opposite trend was observed (Fig. 3b). We also found that knocking out APN reduced the antiapoptotic ability of SK-HEP-1. The proportion of apoptotic cells induced by cisplatin was significantly higher in SK-KO cells than in SK-Con cells (Fig. 3c). Conversely, overexpressing APN in Huh6 led to an increase in the antiapoptotic ability (Fig. 3d).

The in vivo experiment to observe the effect of APN on xenograft tumor growth was conducted in a subcutaneous model. We found that the SK-KO group tumors were significantly smaller than those in the control group, and the weights of the final resected tumors in the SK-KO group were significantly lower than those in the control group (Fig. 3e). Two mice in the control group died during the experimental observation, which may be related to the metastasis of the tumor in the body. Overall, these findings suggested that APN might promote tumor growth and cancer progression in HCC.

### APN exerts functions by activating the ERK signaling pathway

**Fig. 4:**
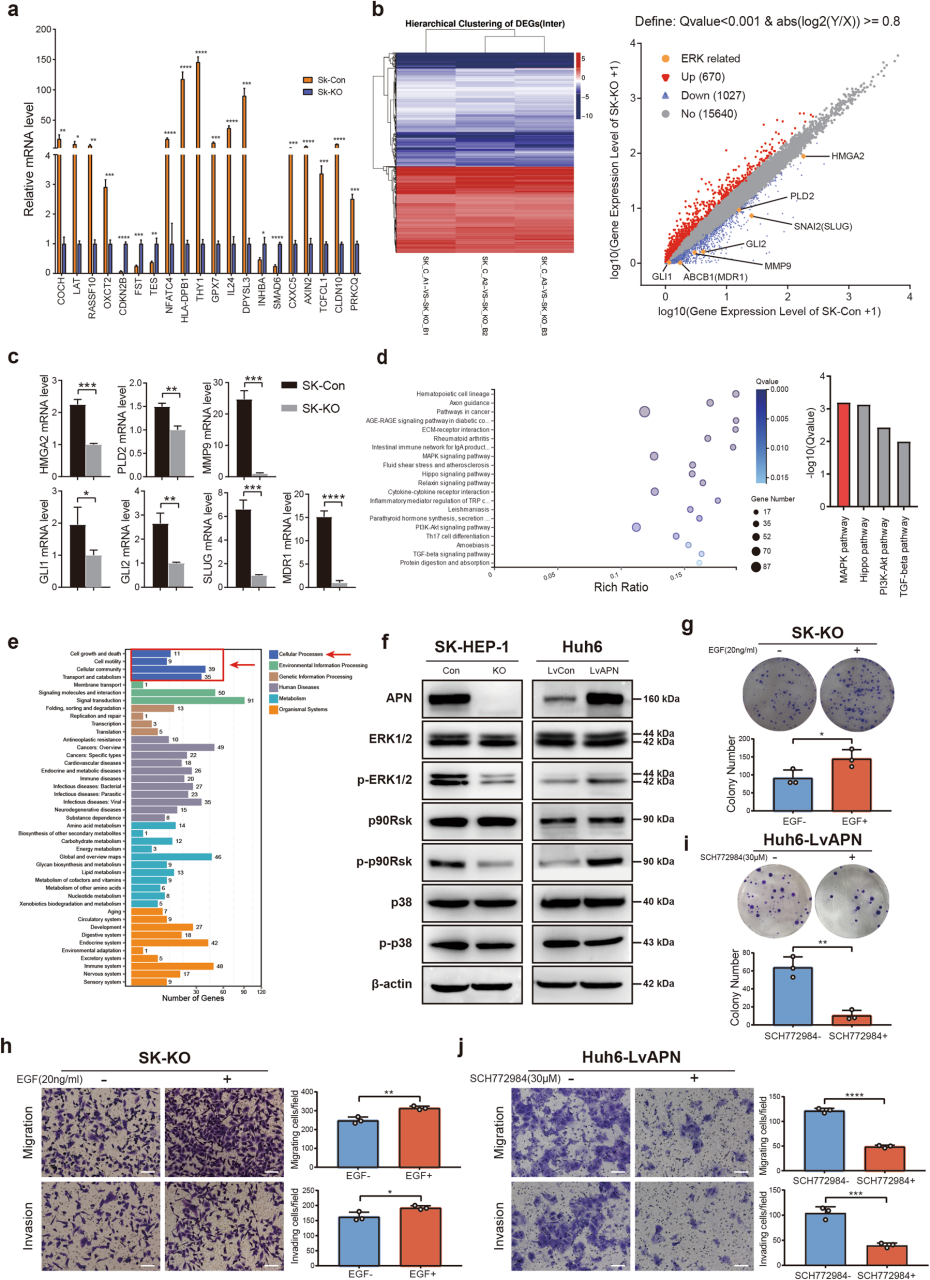
RNA-seq analysis shows that APN regulates biological functions associated with tumor growth and metastasis via ERK signaling pathway. **a** RT-qPCR revealed relative expression of twenty genes that randomly selected. Analysis result of RT-qPCR was consistent with RNA-seq. Mean ± SD, *p < 0.05, **p < 0.01, ***p < 0.001, ****p < 0.0001, Student’s *t*-test. **b** Differentially expressed genes were identified by RNA-seq analysis. Hierarchically clustering showed genes regulated by knockout of APN. Red indicates upregulated genes, and blue indicates gene downregulated genes. Scatter plot showed regulated genes defined by *Q*-value < 0.001 and abs (log2(*Y*/*X*)) ≥ 0.8. **c** Expression of candidate genes regulated by ERK pathway was determined using quantitative RT-PCR in SK-Con and SK-KO cells. Mean ± SD, *p < 0.05, **p < 0.01, ***p < 0.001, ****p < 0.0001, Student’s *t*-test. **d** KEGG pathway enrichment analysis of differentially expressed genes in HCC cells was showed in bubble chart and Histogram. **e** APN-related cellular processes were indicated by analysis based on the RNA-seq data. **f** ERK, p-ERK, p90RSK, p-p90RSK, p38, p-p38, and APN were assayed in modified SK-HEP-1, Huh6, and their control cells by Western blot analyses. **g**, **h** Colony formation assay and transwell migration and invasion assays of SK-KO cells treated with EGF (20 ng/ml). **i**, **j** Colony formation assay and transwell migration and invasion assays of Huh6-LvAPN cells treated with ERK inhibitor SCH772984 (30 μM). Scale bar: 100 μm. Mean ± SD, *p < 0.05, **p < 0.01, ***p < 0.001, ****p < 0.0001; Student’s *t*-test. All quantitative data in the present experiments were performed three times.

To investigate the mechanisms underlying APN-induced HCC metastasis and proliferation, total RNAs from SK-KO and SK-Con cells were subjected to RNA-seq. Twenty differentially expressed genes in the RNA-seq results were randomly selected and analyzed by RT-qPCR. The results showed that the differential expression trends of these genes were consistent with those observed in the RNA-seq data (Fig. 4a). A total of 1027 downregulated and 670 upregulated genes were found to be differentially expressed, with *Q*-value < 0.001 and abs (log_2_(*X*/*Y*)) ≥ 0.8.

Among the differentially expressed genes, we found that several genes related to cancer progression (HMGA2, PLD2, MMP9, GLI1, GLI2, SLUG and MDR1) were expressed at a low level after knocking out APN (Fig. 4b). The expression of these genes in SK-Con and SK-KO cells was verified by RT-qPCR analyses (Fig. 4c). Moreover, previous research suggested that these genes could be directly or indirectly regulated by ERK1/2^27–33^. Since ERK1/2 is widely considered to be functional after being activated via phosphorylation, we speculated that APN might regulate cancer-associated genes downstream of ERK1/2 by modulating the phospho-ERK signaling pathway and then promoting the metastasis and proliferation of HCC.

The KEGG enrichment analysis of differentially expressed genes revealed that the MAPK pathway was significantly altered upon APN knockout (Fig. 4d). A functional analysis of the RNA-seq data showed that there were mainly four cell processes that were altered in the APN knockout cells: cell growth and death, cell motility, cell community, and transport and catabolism (Fig. 4e). These transcriptome changes are consistent with the phenotypes of APN knockout SK-HEP-1 cells, and this consistency validates and supports the viewpoint mentioned above that APN can regulate the metastasis and proliferation of HCC.

To confirm this result, immunoblotting was performed to demonstrate that APN knockout in SK-HEP-1 cells indeed caused ERK1/2 dephosphorylation. Our results showed that APN knockout significantly decreased the phosphorylation protein levels of ERK1/2 and P90RSK, but there was no significant change in the phosphorylation of p38 (Fig. 4f). This result suggested that APN might activate the MAPK pathway by phosphorylation of ERK but not by phosphorylation of p38. In addition to immunoblotting, we carried out transwell and colony formation assays to examine whether APN-mediated HCC metastasis and growth were dependent on the activation of ERK1/2. ERK1/2 serves as a major phosphorylation pathway activated by EGF^34^, and we found that the colony formation and metastatic abilities of SK-KO cells were significantly increased in the absence of EGF (Fig. 4g, h). Following treatment with an ERK inhibitor (SCH772984), Huh6-LvAPN cells showed a decline in colony formation and metastatic abilities (Fig. 4i, j). We speculated that the MAPK/ERK signaling pathway is crucial for APN-mediated HCC growth and metastasis and that ERK plays an important role downstream of APN.

### APN-mediated BCKDK serine 31 phosphorylation during liver cancer progression

**Fig. 5:**
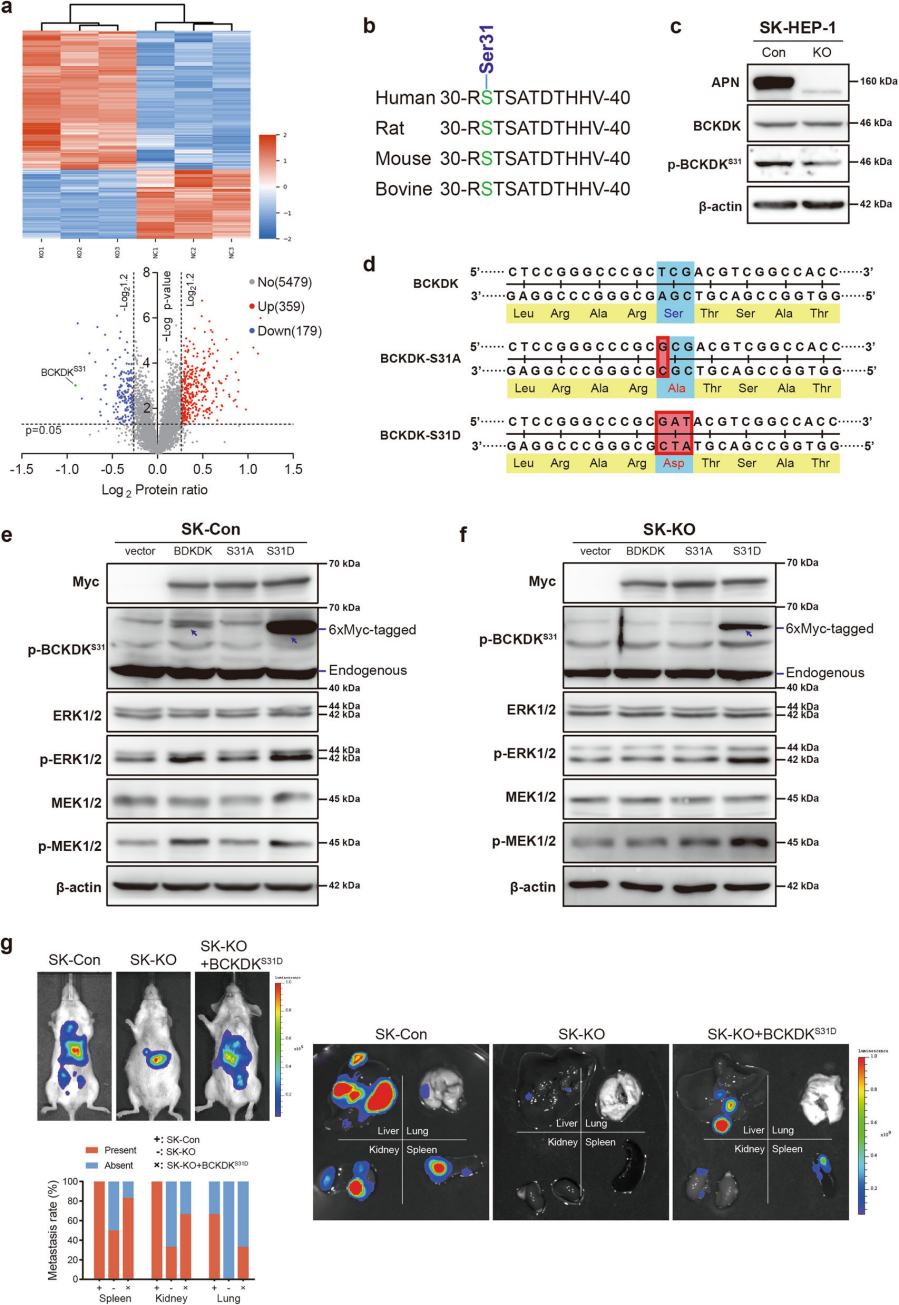
A phosphoproteomic screen identifies BCKDK^S31^ as a candidate APN target during liver cancer metastasis. **a** Hierarchically clustering of differentially expressed phosphoproteins and volcano plot of –log (*p*-value) versus the log_2_ fold change for quantified phosphopeptides. Red and blue indicated up- and downregulated phosphoproteins between SK-Con cells and SK-KO cells, respectively. The protein in green indicated the most significant decrease in phosphorylation. **b** Alignment of the amino acid consensus sequences in BCKDK from different species. **c** Western blotting showed APN, BCKDK, and p-BCKDK^S31^ expression in SK-Con and SK-KO cells. **d** Schematic presentation of the sequencing results of the mutant plasmids. **e**, **f** SK-Con and SK-KO cells were transfected with 6xMyc-tagged control vector, BCKDK, BCKDK^S31A^ and BCKDK^S31D^ plasmid. Western blot of Myc-tag, p-BCKDK^S31^, ERK, p-ERK, MEK, and p-MEK expression in SK-Con and SK-KO cells treated with indicated mutants. 6×Myc tagged and endogenous p-BCKDK^S31^ were indicated, respectively. **g** Representative bioluminescence imaging of liver orthotopic tumor and multiple organ metastasis derived from mouse livers injection with luciferase-labeled SK-Con, SK-KO or SK-KO + BCKDK^S31D^ cells (*n* = 6 in each group). Occurance rates of metastasis in different organs are represented by histogram.

To gain insight into the ERK signaling pathway regulated by APN during HCC progression, SK-KO and SK-Con cells were subjected to a tandem mass tag (TMT) quantitative proteomic analysis. Total peptides were enriched for phosphopeptides using immobilized TiO_2_, resulting in the quantification of 12,145 phosphopeptides from 7256 proteins, of which 6017 peptides have differential phosphorylation levels. Positioning abs (log_2_ (protein abundance ratio)) >log_2_1.2, in the range of *p* < 0.05, 359 peptides with increased phosphorylation levels and 179 peptides with decreased levels were obtained. We identified the phosphorylation of serine 31 (S31) in BCKDK as the most significant decrease in phosphorylated protein after APN knockout (Fig. 5a), and this site on BCKDK appeared as a conserved sequence in multiple species (Fig. 5b). Meanwhile, we generated an antibody that could recognize BCKDK containing phosphorylated S31 with high specificity. This anti-p-BCKDK^S31^ antibody was used to detect SK-Con and SK-KO cells by immunoblotting, and the results were consistent with those of the phosphoproteomic analysis (Fig. 5c).

Since BCKDK is a protein kinase, we sought to verify whether the activation of ERK1/2 mediated by APN is affected by the phosphorylation of BCKDK-S31. To test this hypothesis, we first created two 6×Myc-tagged BCKDK mutant plasmids, BCKDK-S31A and BCKDK-S31D, by using a 6xMcy-tagged normal BCKDK plasmid. Changing serine 31 in BCKDK to alanine (S31A) eliminated the ability of BCKDK-S31 to be phosphorylated, while Ser31→Asp (S31D) mimicked the phosphorylation of S31^25,35^ (Fig. 5d–f).

Subsequently, a series of recombinant plasmids containing 6×Myc-tag were transfected into SK-Con and SK-KO cells, including normal BCKDK, BCKDK^S31A^, BCKDK^S31D^ and control vector. In the APN normally expressed SK-HEP-1 cells (SK-Con), transfection with a plasmid expressing normal BCKDK or BCKDK^S31D^ resulted in a significant increase in ERK1/2 phosphorylation. Transfection with the plasmid expressing BCKDK^S31A^ did not increase the phosphorylation level of ERK1/2 (Fig. 5e). In the APN knockout SK-HEP-1 cells (SK-KO), only BCKDK^S31D^_,_ which mimicked S31 phosphorylated BCKDK can increase the phosphorylation level of ERK1/2 (Fig. 5f). These results indicated that phosphorylation of serine 31 in BCKDK can increase ERK phosphorylation in HCC cells, and this effect was dependent on the presence of APN. Previous studies have shown that BCKDK can phosphorylate MEK1/2^36^, and our experimental results were consistent with this. Our study also showed that, like ERK1/2, BCKDK can only phosphorylate MEK1/2 in the presence of APN (Fig. 5e, f).

We further injected luciferase-expressing SK-Con, SK-KO and SK-KO + BCKDK^S31D^ (SK-KO cells overexpressing BCKDK^S31D^) cells into the left liver lobe of mice. Six weeks after the injection, the mice were sacrificed and examined for tumor growth at the transplantation site and metastasis to other major organs. Compared with the SK-Con mice, the SK-KO mice showed decreased levels of metastasis and tumor growth. However, both of these indicators were significantly higher in the SK-KO + BCKDK^S31D^ group than in the SK-KO group (Fig. 5g). The in vivo results suggested that overexpressing BCKDK^S31D^ in APN knockout SK-HEP-1 cells could reverse the decline in tumor metastasis and the slower growth. Overall, these results provide further evidence that downregulation of APN significantly reduced the phosphorylation of ERK, thereby reducing the rate of HCC tumor development in vivo, whereas overexpression of BCKDK^S31D^ can effectively reverse this phenotype.

### BCKDK directly binds to ERK1/2

**Fig. 6:**
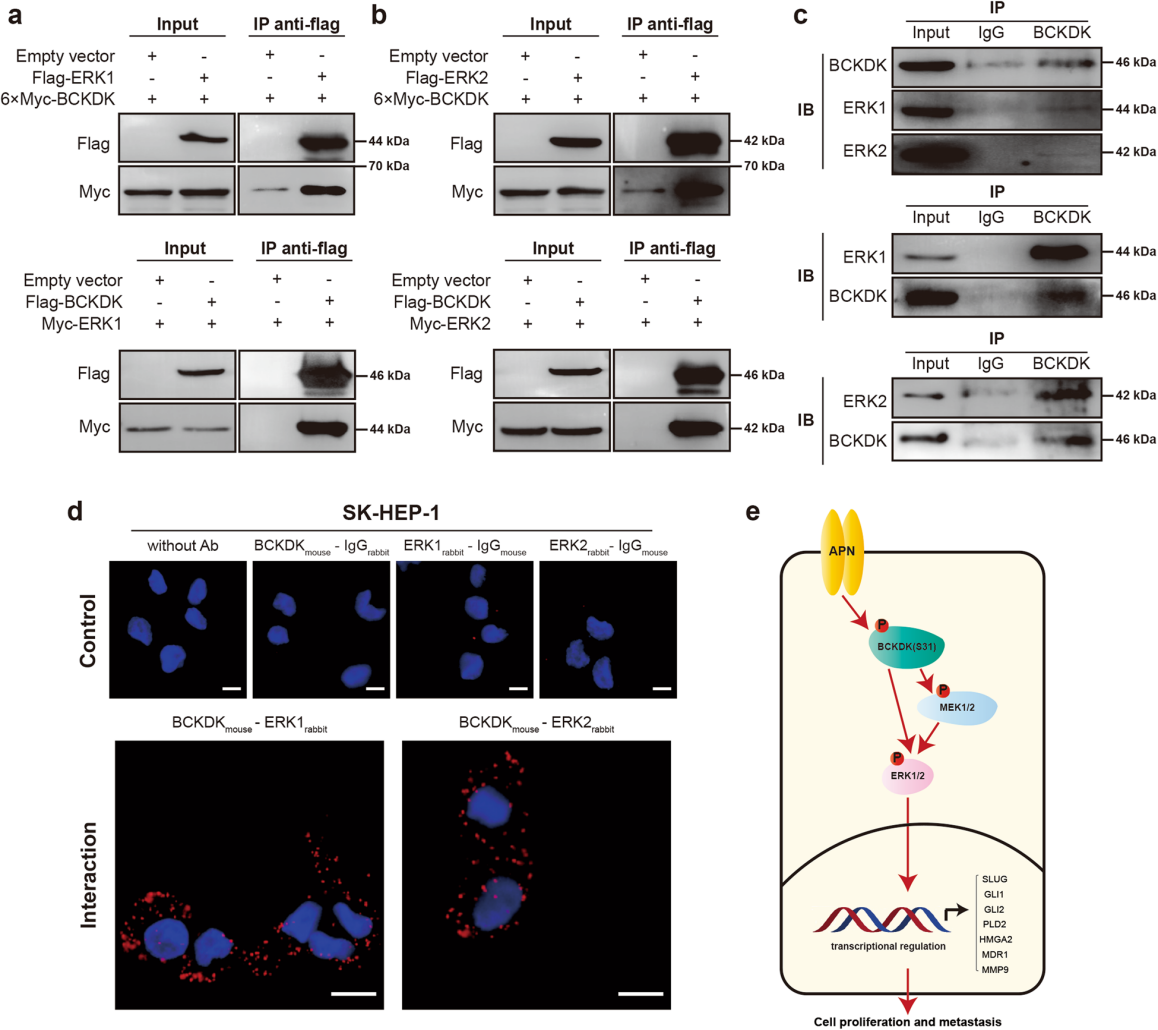
BCKDK acts directly on erk1/2. **a** Co-Immunoprecipitation of Flag-ERK1 with 6×Myc-BCKDK and Co-IP of Flag-BCKDK with Myc-ERK1 in 293T cells. **b** Co-IP of Flag-ERK2 with 6×Myc-BCKDK and Co-IP of Flag-BCKDK with Myc-ERK2 in 293T cells. **c** Endogenous BCKDK and ERK1 (or ERK2) was immunoprecipitated from SK-HEP-1 cells and then probed with anti-BCKDK antibody, anti-ERK1 and anti-ERK2, respectively. The protein levels of BCKDK, ERK1 and ERK2 detected by western blotting. **d** Erk1-BCKDK interactions and ERK2-BCKDK interactions detected by PLA in SK-HEP-1 cells. Cell nuclei were stained blue with DAPI. Red spots indicated the protein interactions. The specificity of PLA was evaluated by omitting two primary antibodies or one of the two primary antibodies. Scale bar: 10 μm. **e** Schematic presentation of the mechanism for APN-driven phosphorylation of BCKDK and downstream regulation of ERK signaling pathway in HCC.

To determine whether BCKDK directly interacts with ERK1 or ERK2, a co-IP assay was conducted to validate the interaction between exogenous BCKDK and ERK1 (or ERK2) in 293T cells. The co-IP results showed that BCKDK could pull down ERK1 (or ERK2) from the cell lysate of 293T cells transfected with related plasmids, and vice versa (Fig. 6a, b). Moreover, endogenous BCKDK also interacted with ERK1 (or ERK2) in SK-HEP-1 cells (Fig. 6c). Moreover, we observed the direct interaction between BCKDK and ERK1 (or ERK2) in cultured HCC cells by a PLA. BCKDK was found to interact with ERK1 significantly in SK-HEP-1 cells, and the negative control evaluated the specificity of the PLA by omitting the two primary antibodies or one of the two primary antibodies. The same interaction was also found between ERK2 and BCKDK. (Fig. 6d) These data suggested that BCKDK could bind to ERK1/2 directly, implicating certain biological significance of this binding during HCC development.

## Discussion

Metastasis and recurrence of cancer are important causes of death and poor prognosis in patients, and >90% of mortality from cancer can be attributed to metastasis^3,37^. Identifying new therapeutic targets, especially those targeting metastasis, is critical to advancing treatment for HCC patients. In this study, we demonstrated that APN is a potent oncogene responsible for HCC metastasis and proliferation. APN mediated the phosphorylation of BCKDK at serine 31, which in turn activated the ERK signaling pathway to promote the progression of HCC (Fig. 6e).

Aminopeptidase N (APN/CD13) is an important transmembrane metalloprotease that regulates multivariate cellular functions by different mechanisms, such as enzymatic cleavage of peptides, degradation of the extracellular matrix, signal transduction, antigen presentation, and many more^6,38^. Expression disorder of APN expression has been shown to promote many kinds of human cancer development^39–41^. In addition, APN is commonly used as a candidate marker for liver cancer stem cells^10,42^, and the APN/CD13^+^ cell population in HCC might be associated with cancer cell chemoresistance^9^. Here, we showed that high expression of APN is associated with larger tumors in HCC patients and a stronger tumor cell metastatic ability than low expression of APN.

Through gain- and loss-of-function experiments, we confirmed that APN positively regulated the migration, invasion, proliferation and antiapoptotic ability of HCC cells in vitro. Furthermore, in liver tumor metastasis models, the APN knockout group exhibited less and smaller intrahepatic metastatic nodules, lower incidence of tumors spreading to other organs outside the liver, and longer experimental animal survival time. In the subcutaneous HCC xenograft tumor experiment, the tumor volumes and weights in the APN knockout group were significantly smaller than those in the control group. Therefore, our results indicate that APN plays a significant role in both HCC metastasis and proliferation.

On the molecular level, our investigation uncovered the role of APN in the metastasis and proliferation of HCC by regulating the MAPK/ERK signaling pathway. As APN knockout markedly decreased ERK1/2 phosphorylation in this study, we thought it served as a regulator to favor ERK1/2 phosphorylation, thus activating MAPK/ERK signaling. The MAPK/ERK signal transduction pathway can be activated in response to a variety of extracellular stimuli, including mitogens, growth factors and cytokines, and researchers believe it is an important target for cancer diagnosis and treatment^43–45^. Overexpression or activation of components of the MAPK/ERK pathway has been reported to result in tumorigenesis, disease metastasis and tumor proliferation in a variety of solid tumors^46–48^. We found that APN was involved in the phosphorylation of the major proteins in the phospho-ERK1/2 pathway downstream of the MAPK signal transduction pathway, rather than the p38 pathway, in HCC cells. In addition, the pro-metastatic and pro-proliferative effects of APN can be offset by treating APN overexpressing HCC cells with an ERK inhibitor and treating APN knockout HCC cells with EGF.

Activated ERK regulates the target protein in the cytoplasm and can also be transported into the nucleus to phosphorylate a variety of transcription factors that regulate gene expression in the nucleus^49–51^. Along with the phosphorylation of ERK1/2 regulated by APN, we found that effector proteins regulated directly or indirectly by ERK1/2 are elevated in HCC cells, including HMGA2^27^, PLD2^28^, MMP9^29^, GLI1^29–31^, GLI2^30,31^, SLUG^27,28,32^, and MDR1^33^. These proteins are important tumor growth and metastasis promoting factors, and this conclusion is consistent with the results of recent studies: in colon cancer, HMGA2 was upregulated by activated ERK1/2 and increased the expression of the transcription factor SLUG, thereby promoting migration, invasion and proliferation^27^; in breast cancer, ERK-mediated SLUG phosphorylation could regulate PLD2 to promote cancer invasiveness^28^; in HCC, phospho-ERK can enhance tumor metastasis and growth by activating the level of GLI1 and MMP9^29^; in human lung fibroblasts, the participation of the mitogen-activated protein kinase kinase kinase 1 (MEKK1)/MEK1/ERK1/GLI-1/GLI-2 and activator protein-1 (AP-1) signaling pathways mediated hypoxia-induced CTGF expression^30^; in colon cancer tissues, SMYD2-OE could upregulate MDR1 expression depending on MEK/ERK/AP-1 signaling pathway activity^33^. Therefore, we speculated that phosphorylation activation of ERK1/2 is crucial for APN-mediated HCC metastasis and growth.

Given the lack of kinase activity, we postulated that APN elevates ERK1/2 phosphorylation in an indirect manner. After phosphoproteomic analyses in this study, the most significant change in SK-KO cells was a decrease in the phosphorylation of branched-chain α-keto acid dehydrogenase kinase (BCKDK) on serine 31. BCKDK is an important regulatory enzyme in branched-chain amino acid (BCAA) catabolism^17,52^. By dephosphorylating the E1 component of the branched-chain α-keto acid dehydrogenase (BCKDH) complex and inactivating it, BCKDK played a key role in the BCAA catabolic pathways^53^. Abnormalities in BCKDK activity often lead to many pathological conditions^15–18^. A previous study suggested that BCKDK enhanced the MAPK signaling pathway by directly phosphorylating MEK and activating downstream ERK to promote the progression of colorectal cancer^36^. However, the mechanism by which abnormal BCKDK activity is produced in the course of cancer remains unclear. Our results showed that normal BCKDK produces the same effect as BCKDK^S31D^ when APN is present. In addition, either a normal BCKDK protein or a S31-nonphosphorylated BCKDK protein (BCKDK^S31A^) cannot induce the phosphorylation of ERK1/2 in APN knockout SK-HEP-1 cells. However, the BCKDK protein, in which the phosphorylation of serine 31 was simulated (BCKDK^S31D^) can phosphorylate and activate ERK1/2 in APN knockout SK-HEP-1 cells, thereby restoring their proliferation and metastatic phenotypes in a liver orthotopic xenograft tumor model. Our Co-IP and PLA experiments showed that BCKDK can directly bind to ERK1 and ERK2. This means that in addition to activating MEK to phosphorylate ERK^36^, BCKDK may also directly phosphorylate ERK1/2 through its kinase activity.

Here, our experiments first showed that APN mediates the phosphorylation of BCKDK on serine 31, which in turn activates ERK1/2 and promotes HCC proliferation and metastasis. BCKDK is a vital tool to assist in the homeostasis of BCAA, including valine, leucine, isoleucine, etc., while APN is capable of producing these BCAAs by enzymatically hydrolyzing its substrate polypeptide^54^. Therefore, the APN-mediated regulation of BCKDK activity in liver cancer may be related to its abnormal enzyme activity affecting BCAA catabolism homeostasis, and we plan to investigate this hypothesis in the future.

In summary, we demonstrated that APN is an important oncogene that contributes to HCC metastasis and proliferation. The APN-mediated promotion of cancer development is achieved by mediating the phosphorylation of BCKDK on serine 31 and then activating the ERK1/2 signaling pathway through the direct action of BCKDK. Our results provide a basis for understanding the mechanisms of HCC progression and may be helpful to identify new biomarkers and therapeutic targets for HCC.

## Supplementary information

Supplemental Materials and methods

Table S1

Table S2

Table S3

Table S4

Table S5
