# Supplementary material for: APN-mediated phosphorylation of BCKDK promotes hepatocellular carcinoma metastasis and proliferation via the ERK signaling pathway
Source: Cell Death Dis. 2020 May 26;11(5):396. doi: 10.1038/s41419-020-2610-1 (PMC7249043; doi:10.1038/s41419-020-2610-1)
Supplement: Supplementary file 1 — Supplemental Materials and methods [file 41419_2020_2610_MOESM1_ESM.docx]

**Supplemental Materials and methods**

**RNA sequencing (RNA-seq)**

**mRNA Library Construction**. DNase I was used to digest double- and single-stranded DNA in total RNA, and then magnetic beads were purified to recover the reaction products, rRNA was removed using the Ribo-Zero method (Illumina, USA). Purified mRNA from the previous step was fragmented into small pieces using fragment buffer at the appropriate temperature. Then, the first-strand cDNA was generated by PCR in the first-strand reaction system, and the second-strand cDNA was also generated. The reaction product was purified by magnetic beads, and then A-Tailing Mix and RNA Index Adapters were added by incubation for end repair. cDNA fragments with adaptors were amplified by PCR, and the products were purified by Ampure XP Beads. Library was being validated for quality control using an Agilent Technologies 2100 bioanalyzer. The above double-stranded PCR product was heated denatured and circularized by splinting the oligonucleotide sequence. Format single strand circle DNA (ssCir DNA) into the final library. The final library was amplified with phi29 (Thermo Fisher Scientific) to prepare DNA nanoball (DNB) with more than 300 copies of one molecule, DNBs were loaded into a patterned nanoarray, and generated single-end 50 bases reads on BGISEQ500 platform (BGI-Shenzhen, China).

**Phosphoproteomic Analysis**

**Sample Preparation.** SDT lysis buffer [4% SDS, 100 mM Tris-HCl pH 7.6, and protease and phosphatase inhibitor tablets (Roche, Basel, Switzerland)] were added to the samples. Lysates were then subjected to sonicate and boiling water bath (15 minutes) followed by centrifuging (14,000 g, 15 min) and supernatant collection. Proteins were quantified using the BCA protein analysis kit（Beyotime Biotechnology, Shanghai, China）, after which the samples were dispensed and stored at -80 ° C.

**Filter-aided sample preparation (FASP Digestion).** 200 μg of protein per sample was incorporated into 30 μl of SDT buffer (150 mM Tris-HCl pH 8.0, 100 mM DTT, 4% SDS). Remove detergent DTT (Sigma-Aldrich) and other low molecular weight components by repeated ultrafiltration (30KD, SartoriusAG, Gottingen, Germany) using UA buffer (150 mM Tris-HCl pH 8.5, 8 M urea). Then 100 μl iodoacetamide [(100 mM IAA (Sigma-Aldrich) in UA buffer] was added to block reduced cysteine residues, and the samples were incubated in the dark for 30 minutes. The filters were washed with 100 μl UA buffer 3 times, then 100 μl of 0.1M triethylammonium carbonate (TEAB) buffer twice. Finally, the protein suspension was digested in 40 μl of 0.1 M TEAB buffer with 4 μg trypsin (Promega, Madison, Wisconsin) overnight at 37 ° C, and the resulting peptides were collected as a filtrate. The peptide content was estimated by UV light spectral density at 280 nm using an extinctions coefficient of 1.1 of 0.1% (g/l) solution that was calculated on the basis of the frequency of tryptophan and tyrosine in vertebrate proteins.

**Tandem Mass Tag (TMT) Labeling and phosphorylated peptide enrichment.** 100 μg of peptide mixture per sample was labeled according to Thermo's TMT Labeling Kit(Eugene, OR, USA) instructions. Each group of labeled peptides was mixed, desalted and dissolved in 200ul loading buffer (30%ACN, 20mg/ml, 2%TFA). Then the peptide solution was transferred to a test tube containing TiO2 and rotated for 2 h. The mixture was then transferred to stage tips, washed twice with 200ul of wash buffer 1 (70% ACN, 0.1% TFA), and sequentially with Elution Buffer 1 (20% ACN, 0.6% ammonia), and Elution Buffer 2 (50% ACN, 1% ammonia), and Elution buffer 3 (40% ACN, 4.5% ammonia). Finally, the enriched phosphopeptides were freeze-dried and dissolved in 0.1% FA.

**Mass Spectrometry (MS) analysis**. Samples were separated using a nanoliter flow rate Easy nLC system and mass spectrometry was performed using a Q Exactive plus mass spectrometer.

**Data Analysis.** This project used a high-resolution mass spectrometer Q Exactive plus (Thermo Fisher Scientific) for quantitative proteomic analysis of TMT. The database used in this project: Uniprot_HomoSapiens_20386_20180905. The mass spectrometry data was a raw file, and the data was identified and quantified using the software Mascot 2.6 (Matrix Science, London, UK) and Proteome Discoverer 2.1 (Thermo Fisher Scientific, Eugene, OR, USA). Bioinformatics analysis of GO and KEGG.
