# Supplementary material for: APN-mediated phosphorylation of BCKDK promotes hepatocellular carcinoma metastasis and proliferation via the ERK signaling pathway
Source: Cell Death Dis. 2020 May 26;11(5):396. doi: 10.1038/s41419-020-2610-1 (PMC7249043; doi:10.1038/s41419-020-2610-1)
Supplement: Supplementary file 2 — Table S1 [file 41419_2020_2610_MOESM2_ESM.docx]

**Supplemental Table S1. Primers for RT-qPCR**

|  | **Genes** | **Forward Sequence (5’-3’)** | **Reverse Sequence (5’-3’)** |  |
| --- | --- | --- | --- | --- |
|  | APN | TTCAACATCACGCTTATCCACC | AGTCGAACTCACTGACAATGAAG | |
|  | COCH | AGAAAACACCCGAGAAGAAAACT | CCAATTCCCAACATTAGAGCCA |  |
|  | LAT | GATGAGGACGACTATCACAACCC | GAAGGCACTGTCTCGGATGC |  |
|  | RASSF10 | CCCCGCAGTGCTATTGCAT | CACGAAGCGCACATTCTCTT |  |
|  | OXCT2 | AACGGCGACCACTTCCTTTT | ACATGGGCACGTTGAAATTGC |  |
|  | CDKN2B | CAACGGAGTCAACCGTTTCG | GTGAGAGTGGCAGGGTCTG |  |
|  | FST | AGGCAAGATGTAAAGAGCAGC | CAGTAGGCATTATTGGTCTGGTC | |
|  | TES | ATGGGCTTAGGTCACGAGC | TCCCACTTTTCGATCCTCTTCA |  |
|  | NFATC4 | CTTCTCCGATGCCTCTGACG | CGGGGCTTGGACCATACAG |  |
|  | HLA-DPB1 | CAGCACCACAACCTGCTTG | CCATTCAGGAACCATCGGACT |  |
|  | THY1 | ATCGCTCTCCTGCTAACAGTC | CTCGTACTGGATGGGTGAACT |  |
|  | GPX7 | CCCACCACTTTAACGTGCTC | GGCAAAGCTCTCAATCTCCTT |  |
|  | IL24 | CTTTGTTCTCATCGTGTCACAAC | TCCAACTGTTTGAATGCTCTCC |  |
|  | DPYSL3 | GACCGTCTCCTTATCAAGGGA | GCATCTGGAAGTGAGTATGGAC | |
|  | INHBA | CCTCCCAAAGGATGTACCCAA | CTCTATCTCCACATACCCGTTCT | |
|  | SMAD6 | GCTACCAACTCCCTCATCACT | CGTACACCGCATAGAGGCG |  |
|  | CXXC5 | CCGAGCGTCGGAACAAGAG | CCACTGCTGCCAAAAGAAGAG |  |
|  | AXIN2 | TACACTCCTTATTGGGCGATCA | TTGGCTACTCGTAAAGTTTTGGT | |
|  | TCF7L1 | TCGTCCCTGGTCAACGAGT | ACTTCGGCGAAATAGTCCCG |  |
|  | CLDN10 | GCATGTAGAGGACTTATGATCGC | TCCGACTTTGGTACACTTCATTC | |
|  | PRKCQ | ATGTCGCCATTTCTTCGGATT | ACATACTCTTTGACGAGCACAG | |
|  | SLUG | CGAACTGGACACACATACAGTG | CTGAGGATCTCTGGTTGTGGT |  |
|  | MMP9 | TGTACCGCTATGGTTACACTCG | GGCAGGGACAGTTGCTTCT |  |
|  | MDR1 | TTGCTGCTTACATTCAGGTTTCA | AGCCTATCTCCTGTCGCATTA |  |
|  | PLD2 | TCGATTTGCCGTTGCCTATTC | GGTCAAGAGACGGTTGAGGTA |  |
|  | GLI1 | AACGCTATACAGATCCTAGCTCG | GTGCCGTTTGGTCACATGG |  |
|  | GLI2 | CTGCCTCCGAGAAGCAAGAAG | GCATGGAATGGTGGCAAGAG |  |
|  | HMGA2 | ACCCAGGGGAAGACCCAAA | CCTCTTGGCCGTTTTTCTCCA |  |
|  | β-actin | CATGTACGTTGCTATCCAGGC | CTCCTTAATGTCACGCACGAT |  |
