# Supplementary material for: APN-mediated phosphorylation of BCKDK promotes hepatocellular carcinoma metastasis and proliferation via the ERK signaling pathway
Source: Cell Death Dis. 2020 May 26;11(5):396. doi: 10.1038/s41419-020-2610-1 (PMC7249043; doi:10.1038/s41419-020-2610-1)
Supplement: Supplementary file 3 — Table S2 [file 41419_2020_2610_MOESM3_ESM.docx]

**Supplemental Table S2. Relationship between APN expression in HCC and clinicopathologic characteristics (n = 90)**

|  | **Characteristics** | **Frequency** | **Percentage** | **P Value** |
| --- | --- | --- | --- | --- |
|  | Sex |  |  | 0.635 |
|  | Male | 80 | 88.9% |  |
|  | Female | 10 | 11.1% |  |
|  | Age (years) |  |  | 0.012* |
|  | ≥60 | 22 | 24.4% |  |
|  | <60 | 68 | 75.6% |  |
|  | TNM stage |  |  | 0.601 |
|  | T1 | 58 | 64.4% |  |
|  | T2 | 28 | 31.1% |  |
|  | T3 | 4 | 4.5% |  |
|  | Tumor diameter (cm) |  |  | 0.020* |
|  | ≥4 | 42 | 46.7% |  |
|  | <4 | 48 | 53.3% |  |
|  | Cirrhosis |  |  | 0.033* |
|  | Positive | 80 | 89.9% |  |
|  | Negative | 9 | 10.1% |  |
|  | HBsAg |  |  | 0.109 |
|  | Positive | 70 | 78.7% |  |
|  | Negative | 19 | 21.3% |  |
|  | HBcAb |  |  | 0.247 |
|  | Positive | 80 | 92.0% |  |
|  | Negative | 7 | 8.0% |  |

**P value was calculated with Mann-Whitney U test or Kruskal-Wallis test. *P<0.05**
