# Supplementary material for: APN-mediated phosphorylation of BCKDK promotes hepatocellular carcinoma metastasis and proliferation via the ERK signaling pathway
Source: Cell Death Dis. 2020 May 26;11(5):396. doi: 10.1038/s41419-020-2610-1 (PMC7249043; doi:10.1038/s41419-020-2610-1)
Supplement: Supplementary file 4 — Table S3 [file 41419_2020_2610_MOESM4_ESM.docx]

**Supplemental Table S3. Primers for site-specific mutations**

|  | **Site** | **Forward Sequence (5’-3’)** | **Reverse Sequence (5’-3’)** |  |
| --- | --- | --- | --- | --- |
|  | BCKDK(S31A) | GCGCTCCGGGCCCGCGCGACGTCGGC | CGCGGGCCCGGAGCGCGAGTGCGGG | |
|  | BCKDK(S31D) | GCGCTCCGGGCCCGCGATACGTCGGC | ATCGCGGGCCCGGAGCGCGAGTGCGGG | |
