# Supplementary material for: APN-mediated phosphorylation of BCKDK promotes hepatocellular carcinoma metastasis and proliferation via the ERK signaling pathway
Source: Cell Death Dis. 2020 May 26;11(5):396. doi: 10.1038/s41419-020-2610-1 (PMC7249043; doi:10.1038/s41419-020-2610-1)
Supplement: Supplementary file 5 — Table S4 [file 41419_2020_2610_MOESM5_ESM.docx]

**Supplemental Table S4. CRISPR sgRNA oligos**

|  | **sgRNA Target** | **Forward Sequence (5’-3’)** | **Reverse Sequence (5’-3’)** |
| --- | --- | --- | --- |
|  | sgAPN-KO1 | CACCCAGTGCGATGATTGTGCACA | AAACTGTGCACAATCATCGCACTG |
|  | sgAPN-KO2 | CACCAGACTTCAACGCCGGCGCCA | AAACTGGCGCCGGCGTTGAAGTCT |
|  | sgAPN-KO3 | CACCATCGCCGTGGCCCGCCGCAA | AAACTTGCGGCGGGCCACGGCGAT |
