# Supplementary material for: APN-mediated phosphorylation of BCKDK promotes hepatocellular carcinoma metastasis and proliferation via the ERK signaling pathway
Source: Cell Death Dis. 2020 May 26;11(5):396. doi: 10.1038/s41419-020-2610-1 (PMC7249043; doi:10.1038/s41419-020-2610-1)
Supplement: Supplementary file 6 — Table S5 [file 41419_2020_2610_MOESM6_ESM.docx]

**Supplementary Table S5. Primary antibodies with indicated concentration for WB, IHC, IP and PLA.**

| Protein | WB | IHC | IP | PLA | Specificity | Product code |
| --- | --- | --- | --- | --- | --- | --- |
| APN | 1:1000 | 1:6000 |  |  | Rabbit | CST, #32720 |
| ERK1/2 | 1:1000 |  |  |  | Rabbit | CST, #4695 |
| p-ERK1/2 | 1:1000 |  |  |  | Rabbit | CST, #4370 |
| ERK1 | 1:1000 |  | 1:100 | 1:400 | Rabbit | Abcam, ab32537 |
| ERK2 | 1:1000 |  |  | 1:400 | Rabbit | Abcam, ab32081 |
| ERK2 |  |  | 1:100 |  | Mouse | Invitrogen, MA1-099 |
| p90RSK | 1:1000 |  |  |  | Rabbit | CST, #9355 |
| p-p90RSK | 1:1000 |  |  |  | Rabbit | CST, #11989 |
| p38 | 1:1000 |  |  |  | Rabbit | CST, #9212 |
| p-p38 | 1:1000 |  |  |  | Rabbit | CST, #9211 |
| BCKDK | 1:1000 |  | 1:100 | 1:200 | Mouse | Santa Cruz, sc-374425 |
| MEK1/2 | 1:1000 |  |  |  | Rabbit | CST, #9122 |
| p-MEK1/2 | 1:1000 |  |  |  | Rabbit | CST, #2338 |
| Flag-tag | 1:1000 |  |  |  | Mouse | CST, #8146 |
| Myc-tag | 1:1000 |  |  |  | Rabbit | CST, #2278 |
| β-Actin | 1:10000 |  |  |  | Mouse | Sigma-Aldrich，A5441 |
